# Thermosensitive Polymer Blend Composed of Poloxamer 407, Poloxamer 188 and Polycarbophil for the Use as Mucoadhesive In Situ Gel

**DOI:** 10.3390/polym14091836

**Published:** 2022-04-29

**Authors:** Namon Hirun, Pakorn Kraisit, Vimon Tantishaiyakul

**Affiliations:** 1 Thammasat University Research Unit in Smart Materials and Innovative Technology for Pharmaceutical Applications (SMIT-Pharm), Faculty of Pharmacy, Thammasat University, Pathumthani 12120, Thailand; pakorn54@tu.ac.th; 2 Center of Excellence for Drug Delivery System, Department of Pharmaceutical Chemistry, Faculty of Pharmaceutical Sciences, Prince of Songkla University, Hat-Yai 90112, Thailand; vimon.t@psu.ac.th

**Keywords:** poloxamer 407, poloxamer 188, polycarbophil, thermosensitive hydrogels, mucoadhesive polymers, Box–Behnken design

## Abstract

Herein, thermosensitive blends of poloxamer 407 (P407)/poloxamer 188 (P188)/polycarbophil (PCB) were developed in terms of maximized content of PCB (a mucoadhesive polymer) and desired temperature-dependent rheological properties of the blends as in situ gelling matrices. Maximizing PCB content while achieving the preferable rheological characteristics was accomplished through the Box–Behnken design. The quantitative effect of the polymer composition in the blends on the thermosensitive characteristics was evaluated using the fitted design model and the corresponding surface plots. The optimized P407/P188/PCB blend (OPT) was the mixture of 20.000, 7.349 and 0.595% (*w*/*w*) of P407, P188, and PCB, respectively. The thermosensitive micellization of OPT was investigated using differential scanning calorimetry which revealed an overlapping double endothermic peak caused by the temperature-induced micellization of pure micelles in co-existence with the micelles with attached PCB. Mixing PCB with the P407/P188 matrix promoted a more intense mucoadhesion of the blend. After incorporating metronidazole, a model hydrophilic drug, into OPT, the temperature-dependent characteristics of the hydrogel did not change. Metronidazole release from OPT was sustained by an anomalous mechanism. This optimal ternary hydrogel benefiting from thermosensitive gelling and mucoadhesive matrix might be used as a viable platform for mucoadhesive in situ gelling drug delivery.

## 1. Introduction

The concept of in situ gelation is recognized as a potential drug delivery platform for local disease treatment of the mucosal cavity [1,2]. Among various in situ gelling systems, great attention has been devoted to thermosensitive hydrogels [2]. In response to changes in temperature, thermosensitive hydrogels can convert from solution to gel (sol-gel) state. Given that the ambient temperature is usually lower than the physiological temperature, this can be used as a trigger for the sol-gel transition. The temperature at which the thermosensitive system presents the sol-gel transition is called the gelation temperature. If the gelation temperature of the thermosensitive hydrogels is close to the physiological temperature, the thermosensitive hydrogels can be administered as a liquid and undergo in situ gelation following injection into the body cavity [3]. Therefore, an optimal thermosensitive matrix is a low viscosity fluid around room temperature and forms gel at elevated temperatures after being administered to the body [4,5,6]. These characteristics make the thermosensitive matrix suitable for injection. Furthermore, sol-gel transition near physiological temperatures could prevent the initial burst release of the active compound inside the vehicle and then provide controlled drug release to the target site [6,7,8].

Poloxamers are a class of amphiphilic triblock copolymers composed of a central hydrophobic poly(propylene oxide) (PPO) block and two terminal hydrophilic poly(ethylene oxide) (PEO) blocks. Among divergent types of poloxamers, poloxamer 407 (P407) with the triblock structure of PEO_100_-PPO_65_-PEO_100_ has attracted great interest for potential applications in drug delivery owing to its biocompatibility and thermosensitive behavior [3,9]. Due to hydrogen bonding between the polymer blocks and water molecules, P407 is liquid at low temperatures [10]. The hydrogen bonds destabilize upon heating, and the self-assembly of P407 chains allows them to arrange into micelles with a hydrophobic PPO core and a hydrophilic PEO corona at a critical micelle temperature [9,10]. At a certain concentration of P407, the liquid turns into gel at high temperatures because of the ordered packing of micelles and micelle entanglements [10,11]. Unfortunately, the application of the thermosensitive gels based on plain P407 as the in situ gelling vehicle is limited due to their low gelation temperatures, which hinder administration [8,11].

Polymer blending is considered a potential strategy to regulate the gelation behavior of P407. In addition, polymer blending is an economically favorable method for developing new and improved polymeric materials as it avoids the use of toxic precursors and complicated synthetic procedures [3]. Combining two or more polymers can produce intriguing polymeric platforms, and compositional changes can modulate their properties on purpose [12]. Therefore, understanding the impact of various polymers and their concentration on the concerning characteristics of the polymer blend is essential.

Recently, previous works in our lab have shown that introducing secondary or tertiary species to the matrix can control the temperature-dependent gelation of thermosensitive polymeric solutions [3,13]. The thermosensitive response of P407 is mainly attributed to the temperature-dependent hydrophobic/hydrophilic balance that drives self-assembling and micelle aggregation [6,10]. If the influence of the additives on the focused attribute is understood, the thermosensitive phenomena of P407 solutions might be tailored as required by combining P407 with the suitable polymeric excipients at a precise composition. Using a poloxamer mixture is one of the potential approaches for modifying temperature-dependent features without synthesizing new molecules [14]. The mixtures of poloxamers with variable PPO and/or PEO block lengths may modify the micellization and gelation of individual poloxamers [15,16]. Besides the poloxamer mixture, the existence of some mucoadhesive polyacrylates, such as poly(acrylic acid) and poly(acrylic acid) derivatives has also been reported to affect the gelation temperature of the P407-based vehicle [17,18]. Although the addition of a mucoadhesive polymer improved mucoadhesiveness, the association between the acrylic backbone and the poloxamer chain might diminish the flowability and syringeability of P407-based matrices in liquid form [19]. Most previous works have been focused on the thermosensitive behavior of binary blends containing P407. It was interesting to examine the effect of adding another poloxamer together with a polyacrylic acid polymer into the P407-containing solution. More importantly, understanding the relationship among components in the ternary polymeric mixtures could be valuable in creating a thermosensitive gel with the desired property. However, these multiple factors may affect the thermosensitive nature of the gels.

Response surface methodology (RSM) is a scientific approach for systematically determining the relationship between multiple causal factors and response variables through experiments and statistics [20]. It is also possible to predict the optimal level of independent factors required for a certain response. RSM is thus a systematic and effective method for studying the effects of multiple factors and determining an optimum preparation. The Box–Behnken design, which possesses good symmetry and rotatability, is a very effective RSM design [21]. When three independent variables are examined, the Box–Behnken design gives information on the effect of experimental variables in fewer necessary runs than other designs [20,22]. Therefore, the Box–Behnken design is an efficient and cost-effective technique for analyzing the variable impact involved in a multicomponent system [20,22].

Herein, the ternary polymeric blends composed of P407, another poloxamer and polyacrylic derivative were prepared and investigated. Poloxamer 188 (P188), of which triblock PEO_76_-PPO_29_-PEO_76_ structure possesses a dissimilar PPO block length compared with P407 [16], was selected as the polymer additive. Polycarbophil (PCB), which is a homopolymer of poly(acrylic acid) chains crosslinked with divinyl glycol, was also used for preparing the ternary polymer blends. PCB has good adhesion properties and is a safe and non-allergenic excipient for skin and mucous surfaces [18]. The Box–Behnken design was carried out to evaluate how each component and its composition in the blends affected the thermosensitive attributes of these systems for the desired purpose. The ternary polymer gel was tuned for the highest PCB content and desired temperature-dependent rheological properties: appropriate low complex viscosity at low temperature and suitable gelation temperature. This would give the hybrid benefits from the in situ gelling and mucoadhesive matrix. Then, the thermal and mucoadhesive properties of the optimum ternary polymeric blend were examined. Finally, the obtained optimum system was incorporated with metronidazole (MTZ), a model hydrophilic antimicrobial drug, and then characterized in terms of temperature-dependent rheological characteristics and in vitro drug release.

## 2. Materials and Methods

### 2.1. Materials

P407 (Kolliphor^®^ P407), P188 (Kolliphor^®^ P188), MTZ, monobasic potassium phosphate and porcine gastric mucin were all purchased from Sigma-Aldrich (Saint Louis, MO, USA) and used as received. PCB (Noveon^®^ AA-1) was manufactured by Lubrizol Company (Wickliffe, OH, USA) and kindly supplied by Namsiang Co., Ltd. (Bangkok, Thailand). Sodium hydroxide was obtained from Merck (Merck KGaA, Darmstadt, Germany). All other chemicals used were of analytical grade and used as received.

### 2.2. Sample Preparation

The single P407 or P188 solution was prepared by dispersing the required weight of polymer powder in cold distilled water with vigorous stirring according to the previously described ‘cold method’ [3]. The PCB solution was prepared by dissolving the required amount of PCB powder in distilled water and stirring it with a magnetic stirrer until it became transparent. All of the samples were stored in the refrigerator.

Fifty mL of the aqueous ternary mixtures containing P407, P188, and PCB at concentrations in the range listed in Table 1 were prepared. First, PCB was dispersed by adding the required amount of PCB to 25 mL of cold distilled water. The PCB dispersion was then stirred using a magnetic stirrer until a clear solution was obtained. After that, the desired amount of P188 was dispersed into the PCB solution and constantly stirred in an ice bath until the mixture became homogenous. Then, the desired quantity of P407 was dispersed in the blend of PCB and P188. Subsequently, cold distilled water was added to the dispersion to produce the final weight of 50 g. Finally, the ternary polymeric mixture was stirred in the ice bath until all components were completely homogeneous. The binary mixtures were also prepared according to the preceding steps without adding the polymeric component that was not needed.

For preparing the polymeric sample containing 10% (*w*/*w*) mucin, the required quantity of the mucin was dispersed in the polymeric dispersion prior to adjusting the sample weight to the necessary final weight. The aqueous mucin dispersion was prepared by dispersing mucin in water to obtain 10% (*w*/*w*) mucin in water.

To prepare the polymeric matrix containing 0.75% (*w*/*w*) MTZ, the required amount of MTZ was added to the polymeric dispersion prior to adjusting the total weight of the sample to the final weight. An aqueous 0.75% (*w*/*w*) MTZ solution (MTZ solution) was prepared by dissolving the appropriate amount of MTZ into the distilled water. All of the samples were refrigerated.

### 2.3. Design and Optimization of Polymeric Blends

The Box–Behnken design was carried out to design the preparations of polymeric mixtures with various compositions. A total of 17 runs, three levels, and three factors with five center points were produced with Design-Expert^®^ software (version 13; Stat-Ease, Inc., Minneapolis, MN, USA) [20]. The data analysis was employed to evaluate the effect of the concentration of polymers (P407, P188 and PCB) on the measured temperature-dependent rheological properties (complex viscosity and gelation temperature). The experiment field should not be too large, as this will lead to nonrealistic experiments, nor should it be too small, far from an optimal region [22]. According to a preparatory study, the high concentration of PCB in the mixtures gave highly viscous samples. In addition, the levels of the independent variables were screened and chosen based on the preliminary tube inversion experiments, which were carried out to obtain the samples with the phase transition temperature in the range of 20 °C to 40 °C. The levels of the independent variables as well as their coded values (+1, 0, −1) are listed in Table 1. The gelation temperature (Y_1_) and complex viscosity (Y_2_), which were determined using a temperature sweep test, were used as the dependent responses. Design-Expert^®^ software was used to compare several statistical parameters to the best-fitting mathematical model. Model fitting was performed by analyzing the relationship between the independent parameters and each response in a random order. Analysis of variance (ANOVA) was used to determine the statistical significance of the model derived from the Box–Behnken design, with a *p*-value of less than 0.05 being considered significant [23]. To optimize the level of the factors affecting prospective responses, a statistical experimental design based on Box–Behnken design was used under the constraints listed in Table 1. The optimal blend was chosen based on the maximum desirability value reported by Design-Expert^®^ software. The optimal preparation was then utilized to determine the prediction accuracy, which was expressed as the percentage error between the experimental and predicted values [24].

### 2.4. Measurement of Gelation Temperature and Complex Viscosity

The gelation temperature and temperature-dependent complex viscosity were assessed using the temperature sweep test [3,25]. The dynamic rheological properties were analyzed using a HAAKE MARS 40 rheometer (ThermoFisher Scientific, Bremen, Germany) equipped with a parallel plate geometry (60 mm in diameter and a gap of 0.5 mm) and a Peltier system for temperature control. The temperature dependence of the complex viscosity and the dynamic moduli including storage modulus (G′) and loss modulus (G″) were determined by oscillation temperature sweeps from 5 to 45 °C with a heating rate of 1 °C/min at a frequency of 1 Hz. All oscillatory tests were performed within the linear viscoelastic range. The temperature at which the G’ and G” curves intersect is referred to as the gelation temperature [3,25]. All temperature sweep tests were performed in triplicate.

### 2.5. Differential Scanning Calorimetry (DSC)

The DSC analyses were carried out using a Mettler Toledo DSC 3+ model (Mettler-Toledo, Viroflay, France) with IntraCooler and nitrogen as a purge gas. The sample was placed in a 160 μL aluminum pan with a pierced lid. Temperature scans were performed from 5 to 45 °C at a heating rate of 1 °C/min.

### 2.6. Mucoadhesive Analysis

The HAAKE MARS 40 rheometer with a 60 mm parallel plate geometry was used to assess the dynamic viscoelasticity of polymeric systems in the presence or absence of mucin as a function of angular frequency at 37 °C. In order to obtain a highly sensitive reading, a double cone sensor system (diameter of 60 mm, cone angle of 2°) was used for the mucin solution which possessed low viscoelasticity [8]. All measurements were performed in three replicates. The rheological synergism parameter (Δ*G*′), which reflects the interaction between the polymeric systems and the mucin, was determined using the following equation [8,26]:

(1) $$\Delta G^{\prime }={G^{\prime }}_{mix}-\left({G^{\prime }}_{m}+{G^{\prime }}_{p}\right)$$

where *G*′*_m_*, *G*′*_p_*, and *G*′*_mix_* were the elastic moduli at 10 Hz for the mucin solution, the polymeric system, and the mixture of polymeric system and mucin, respectively.

For the purpose of comparing Δ*G*′ of distinct samples, the Δ*G*′ value of each sample was normalized with respect to *G*′ values of each polymer system and mucin to calculate the relative rheological synergism (Δ*G*′*_relative_*) as demonstrated in the following equation [27]:

(2) $$\Delta {G^{\prime }}_{relative}=\frac{\Delta G^{\prime }}{{G^{\prime }}_{m}+{G^{\prime }}_{p}}$$

### 2.7. In Vitro Release Study

Evaluation of in vitro drug release was performed by using the membraneless diffusion approach, which enables discerning formulation factors while allowing direct contact between the gel and the release medium [8,28]. The in vitro release experiments were performed in a water bath shaker at 37 °C and at a speed of 40 rpm [8]. A 2 g sample was placed in a flat-bottomed vial and allowed to form a gel. The release medium, 15 mL of pre-warmed phosphate buffer pH 7.4, was then gently poured over the gel surface. At predefined time intervals, 2 mL of the release medium was taken from the vial and replaced with 2 mL of the fresh medium. Phosphate buffer pH 7.4 (USP 43) was prepared by placing 50 mL of 0.2 M monobasic potassium phosphate solution and 39.1 mL of 0.2 M sodium hydroxide solution in a 200 mL volumetric flask, followed by adding water to the volume. The amount of released MTZ was analyzed by using a UV spectrophotometer (UV-1800, Shimadzu, Tokyo, Japan) at a wavelength of 320 nm [8]. The calibration curve (R^2^ ≥ 0.99) was prepared using MTZ concentrations in the range of 2–20 µg/mL (Figure S1 in Supplementary Materials). Each in vitro drug release study was carried out in triplicate.

To better understand the MTZ release mechanism, the mathematical models of drug release were fitted to the experimental data using a DDSolver program [29]. The following mathematical models were applied: Higuchi, Hixson–Crowell and Korsmeyer–Peppas models [30].

The Higuchi model is given by:

(3) $$Q_{t}=K_{H}t^{0.5}$$

where *Q_t_* is the percentage of MTZ released at time *t*, *K_H_* is the release constant of Higuchi.

The Hixson–Crowell model is described by:

(4) $$Q_{t}=100\left[1-{\left(1-K_{HC}t\right)}^{3}\right]$$

where *K_HC_* represents the Hixson–Crowell release rate constant.

The Korsmeyer–Peppas model is described by:

(5) $$Q_{t}=K_{KP}t^{n}$$

where *K_KP_* is the Korsmeyer–Peppas release rate constant. The release mechanism is given by the release exponent (*n*) derived from the analysis of the first 60% of the release curve.

### 2.8. Statistical Analysis

Statistical analysis was performed using IBM SPSS Statistics version 22 for Windows (IBM Corp., Armonk, NY, USA). Statistical comparison between two groups of the samples was performed by using the Student’s *t*-test. The results were considered to be significantly different at a *p*-value < 0.05.

## 3. Results and Discussion

### 3.1. Design and Optimization of P407/P188/PCB Blends

#### 3.1.1. Polymer Type and Concentration Range Affecting Gelation Temperature

The essential aspect for in situ thermosensitive hydrogels is gelation in the desired temperature range. For in situ gelling matrices, the gelation temperature should be between 30 and 36 °C [3]. In this study, the optimum gelation temperature was narrowed to 33–36 °C to ensure that the gelling liquid could be administered into a small body cavity and that it would gel afterward [31]. To evaluate the impact of the independent variables on the gelation temperature, the Box–Behnken design was used. The Box–Behnken design is quite rotatable, and this approach can be used for evaluating main, interaction, and quadratic effects.

The quadratic model prevailed over other models for the gelation temperature; this quadratic model was found to be significant (F-value = 44.37, *p*-value < 0.0001). The relationship between the gelation temperature and the independent variables was represented by the following coded equation.

Y_1_ = **31.54** − **2.84X_1_** + **2.29X_2_** + 0.0442X_3_ + 0.4125X_1_X_2_ + 0.7658X_1_X_3_ − **1.32X_2_X_3_** − 0.0567X_1_^2^ − **3.85X_2_^2^** – 0.1684X_3_^2^ (6)

**Figure 1:**
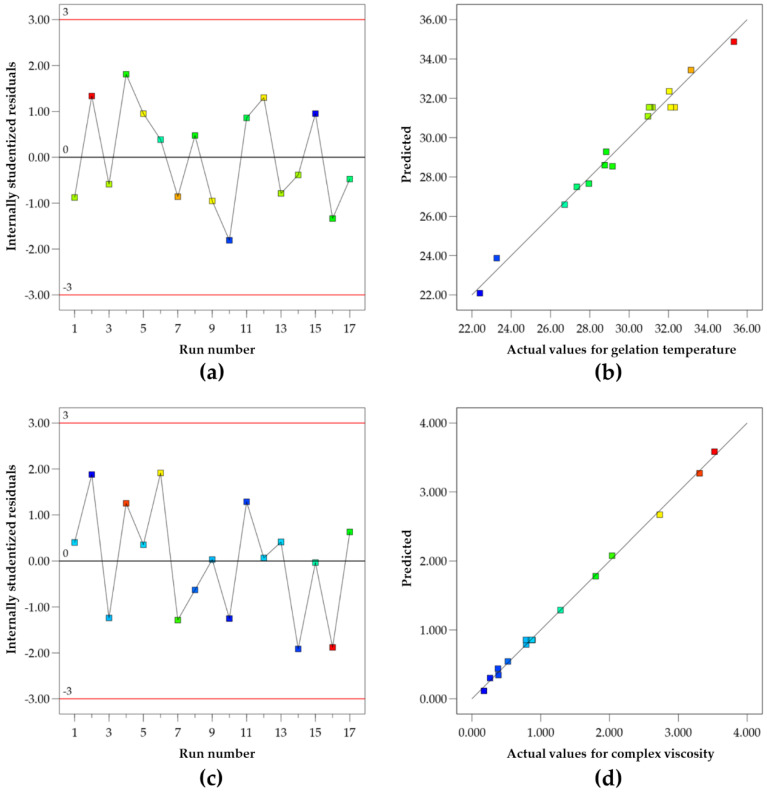
The residual plot between run number and internally studentized residuals for gelation temperature (**a**) and complex viscosity (**c**), and plot of predicted versus actual values for gelation temperature (**b**) and complex viscosity (**d**).

For the derived quadratic model, the Predicted R² of 0.8473 was in reasonable agreement with the Adjusted R² of 0.9828 as the difference is less than 0.2 [32]. The adequate precision of the model was 24.8462. The ratio value of the adequate precision was greater than 4, indicating an adequate signal [22]. This model can be used to navigate the design space. The *p*-value of the lack-of-fit test for this equation was statistically insignificant (*p*-value > 0.05), indicating that the derived quadratic model fitted the data well. The reliability of the dependent variable can be confirmed by the corresponding residual plot between the run number and the internally studentized residuals [20]. As shown in Figure 1a, the vertical distribution of the internally studentized residuals was random scattering and all data points were found to be within the limits of a 95% confidence interval. As illustrated in Figure 1b, the gelation temperatures of all experimental runs varied between 22.41 and 35.33 °C. A uniform distribution of data points around the mean of the response variable implies that the model is acceptable. The proposed equation has a good degree of correlation between the predicted and actual experimental data throughout the design space.

**Figure 2:**
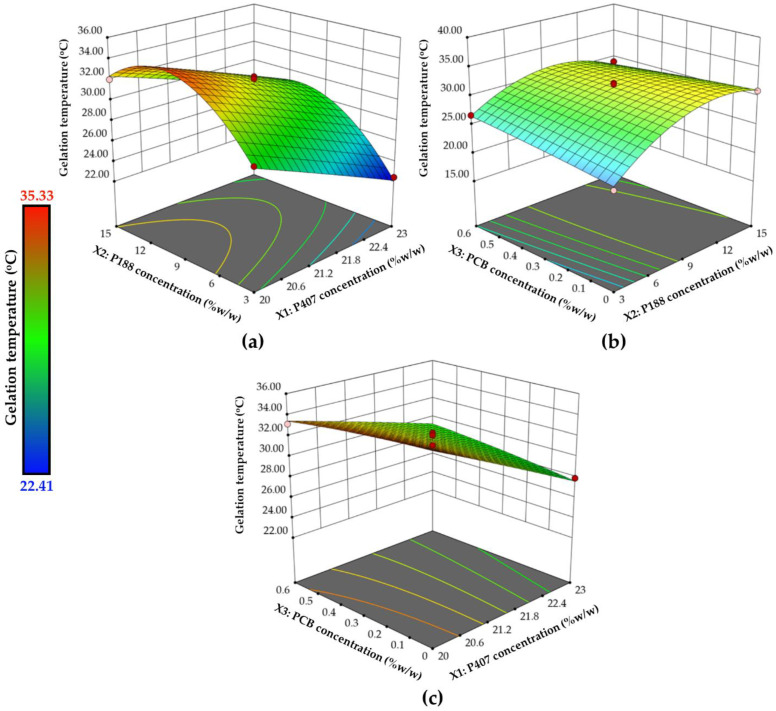
The 3D-response surface plots show the impact of different independent variables on the gelation temperatures; (**a**) effect of X_1_ and X_2_ at the mid-level of X_3_, (**b**) effect of X_2_ and X_3_ at the mid-level of X_1_, and (**c**) effect of X_1_ and X_3_ at the mid-level of X_2_.

In Equation (6), terms written in bold letters are significant. The positive and negative signs that appear before each term show whether the term has a positive or negative effect on the response [23,32]. Significant linear terms included X_1_ and X_2_. A negative value for the coefficient of term X_1_ suggested that an increase in P407 concentration may decrease the gelation temperature. The gelation of P407 is thought to be due to the ordered packing of micelles and inter-micellar entanglements [10]. As the concentration of P407 was increased, the number of P407 chains that were available for micelle formation and subsequent ordered packing increased. Therefore, an increase in P407 concentration favored temperature-induced gelation and caused a lower gelation temperature. The positive coefficient observed for P188 concentration reflected that the gelation temperature of the mixtures tended to shift to a higher value with the increase of P188 concentration. However, significant higher order terms involving X_2_ (the interaction term X_2_X_3_ and the quadratic term X_2_^2^) were found with negative coefficients. The interaction effects of factors on the variability of gelation temperature as well as the impacts of two factors on responses simultaneously are shown as three-dimensional surface plots (Figure 2). Curvature surface was observed in the three-dimensional surface plot involving the effect of P188 concentration (Figure 2a,b), revealing the complexity of the interplay of components. If the concentration of other polymer components was fixed, the gelation temperature increased with an increase in P188 concentration at the low P188 concentration range, but the gelation temperature decreased with further increment in P188 at the high concentration side.

The gelation of poloxamer block copolymers is mainly attributed to the ordered assembly of micelles. The ordered packing of micelles in a mixed solution comprising different poloxamers is determined by the incorporation of block copolymer chains with different hydrophilic-lipophilic block compositions. It has also been reported that the gelation temperature of the poloxamer 238/poloxamer 235 mixture was increased with respect to the gelation temperatures of the parent poloxamers upon the addition of small amounts of poloxamer 238 [15]. Although poloxamer 238 and poloxamer 235 have the same hydrophobic PPO part, the length of hydrophilic PEO blocks of poloxamer 238 is longer than that of poloxamer 235. The longer PEO blocks of poloxamer 238 could intrude on the intermicelle organization, shifting the gelation temperature to higher temperatures [15]. The difference in the hydrophilic and hydrophobic blocks found in P407 and P188 structures also affected the gelation temperature of the mixtures. The effect of P188 at low concentration side is in good agreement with the results obtained by Yuan et al., (2012) who reported an increasing trend in gelation temperature by adding a low to intermediate quantity of P188 in the mixture [33]. The heat-induced gelation of poloxamer solution is the consequence of the ordered packing of poloxamer micelles consisting of swollen PEO corona and a dehydrated PPO core. Poloxamer solution with a high proportion of hydrophobic PPO has low gelation temperature while containing high hydrophilic PEO content caused high gelation temperature [34]. P188 is more hydrophilic than P407; P188 is composed of a higher ratio of PEO/PPO compared with P407. The addition of a small amount of P188 can only change the PEO proportion in the mixed polymer solutions, leading to an increase in the gelation temperature [34]. However, the presence of P188 at relatively high concentration may give rise to the development of P188 micellization in the mixture [34,35]. Apart from the alteration of PEO/PPO ratio in the mixed solution, further increment in P188 concentration was thought to correspond to the formation of P188 micelles that cooperated in the construction of the gel. Therefore, the gelation temperature decreased with further increment in P188 concentration.

#### 3.1.2. Polymer Type and Concentration Range Affecting Complex Viscosity

The in situ thermosensitive hydrogels should be an injectable liquid that can be administered at room temperature (around 20–25 °C) [36]. The complex viscosity of the gelling liquid is indicative of the formulation mobility. Specifically, the gelling liquid with low complex viscosity (<2 Pa s) at a temperature lower than the gelation temperature may be administered easily [4]. According to the gelation temperature of all experimental runs, all thermosensitive hydrogels were liquid at 20 °C. Therefore, the effect of independent variables on the complex viscosity at 20 °C (Y_2_) was also investigated. The appropriate model for the complex viscosity at 20 °C was the quadratic model with a *p*-value < 0.0001 and F-value = 502.57. The relationship between this dependent variable and the independent variables was represented by the following coded Equation (7).

Y_2_ = **0.8557** + **0.4343X_1_** + **0.1842X_2_** + **1.30X_3_** + 0.0615X_1_X_2_ + **0.3194X_1_X_3_** + **0.1160X_2_X_3_** + 0.0522X_1_^2^ + **0.1919X_2_^2^** + **0.6227X_3_^2^** (7)

The lack of fit *p*-value for this equation was statistically insignificant (*p*-value > 0.05). The values of the Predicted R² and the Adjusted R² were 0.9804 and 0.9965, respectively. As shown in Figure 1c, the vertical distribution of the internally studentized residuals was in line from top to bottom in the entirely randomized runs. This indicates that all data fell within a 95% confidence interval. The plots of the actual and predicted complex viscosity are shown in Figure 1d. As can be seen, the observed and predicted values of the response are highly correlated.

**Figure 3:**
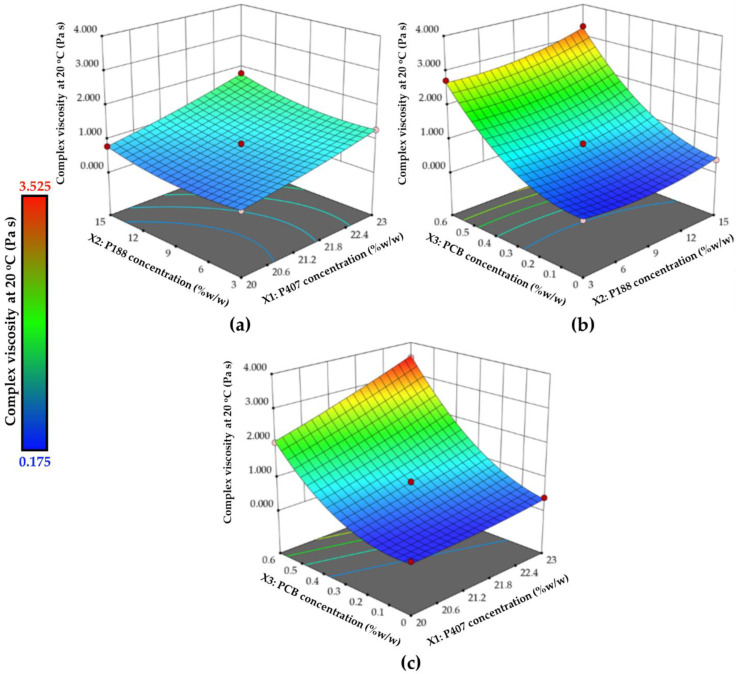
The 3D-response surface plots showing the impact of different independent variables on the complex viscosity at 20 °C; (**a**) effect of X_1_ and X_2_ at the mid-level of X_3_, (**b**) effect of X_2_ and X_3_ at the mid-level of X_1_, and (**c**) effect of X_1_ and X_3_ at the mid-level of X_2_.

Most of the terms in Equation (7) are significant, as indicated in bold letters. All linear terms (X_1_, X_2_ and X_3_) and two quadratic terms (X_2_^2^ and X_3_^2^) are significant, and the positive coefficients of these terms show their positive impact on the complex viscosity. In addition, the interaction terms involving X_3_ (X_1_X_3_ and X_2_X_3_) were also significant. The three-dimensional surface plots in Figure 3 depict the variability of complex viscosity when two factors are changed simultaneously. All polymer components showed a positive effect on the complex viscosity. The increase in complex viscosity, which was caused by increasing polymer concentration, was observed. Despite the absence of actual crosslinking in the sample in the solution stage, the entangled polymer chains can form a viscous liquid. The larger concentrations increased the likelihood of chain interactions (entanglements and rearrangements), resulting in higher complex viscosity [37]. The high positive coefficient values of the terms associated with PCB concentration as well as the predominant effect of PCB concentration on the response observed in the three-dimensional surface plots (Figure 3b,c) indicated that embedding PCB into the mixture of P407 and P188 affected the complex viscosity of the formulation.

#### 3.1.3. Optimization of P407/P188/PCB Blend

As previously stated, the appropriate thermosensitive hydrogel with a high PCB concentration may be selected to achieve a gelation temperature within the optimum temperature range and a liquid with a suitable low complex viscosity. In this study, the optimal range of gelation temperature was set to 33–36 °C. The constrain for complex viscosity at 20 °C was set to less than 2 Pa s. To obtain the optimal thermosensitive hydrogel with a high mucoadhesive polymer concentration, an additional criterion for selection was the highest possible PCB content in the thermosensitive hydrogel. The optimized levels of the independent variables were predicted using numerical optimization. The numerical optimization was performed using the desirability approach. The target parameters included gelation temperature, complex viscosity, and PCB concentration; and the related desirability constraints are shown in Table 1. The thermosensitive hydrogel containing 20.000% (*w*/*w*) P407, 7.349% (*w*/*w*) P188, and 0.595% (*w*/*w*) PCB with the highest desirability of 0.992 met the requirements for an optimal preparation. The optimal thermosensitive hydrogel composed of 20.000% (*w*/*w*) P407, 7.349% (*w*/*w*) P188, and 0.595% (*w*/*w*) PCB was then prepared and this optimal thermosensitive hydrogel was subsequently referred to as OPT. The experimental data were determined to confirm the accuracy and reliability of the predicted optimal preparation [38]. The predicted and experimental values of the responses are shown in Table 2. The results indicated that the mean experimental values of all responses were close to the predicted values and fell within the 95% confidence interval. A comparison of the predicted and experimental values can be used to assess accuracy. The percent error of the prediction was also calculated using the following formula [24]:

(8) $$\mathrm{Percent}\;\mathrm{error}\;\mathrm{of}\;\mathrm{the}\;\mathrm{prediction}\;=\frac{\left(\mathrm{experimental}\;\mathrm{value}-\mathrm{predicted}\;\mathrm{value}\right)}{\mathrm{experimental}\;\mathrm{value}}\times 100$$

The percent error of the prediction was 1.35% and −0.66% for the gelation temperature and the complex viscosity, respectively. The comparison of the predicted value and the experimental value revealed that the prediction by Design-Expert^®^ had a low percentage of error; the values of these percent errors of the prediction were within the 20% acceptability limit [24].

### 3.2. Thermal and Mucoadhesive Properties of the Optimized Polymeric Blend

#### 3.2.1. DSC Analysis

**Figure 4:**
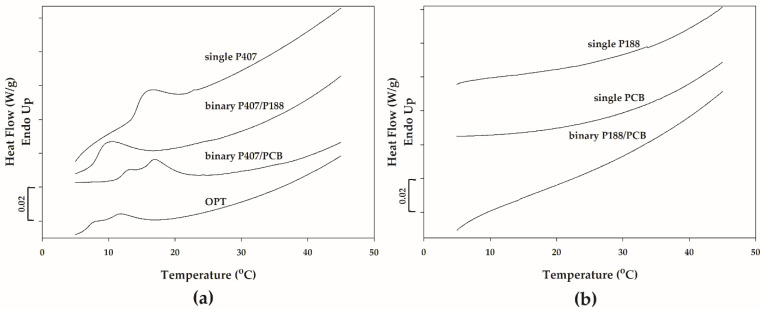
DSC thermograms of (**a**) OPT, 20.000% P407 (single P407), the mixture of 20.000% P407 and 7.349% P188 (binary P407/P188) and the mixture of 20.000% P407 and 0.595% PCB (binary P407/PCB), and (**b**) 7.349% P188 (single P188), 0.595% PCB (single PCB) and the mixture of 7.349% P188 and 0.595% PCB (binary P188/PCB).

DSC is a valuable tool for studying temperature-induced transitions in thermosensitive polymer solutions [35]. The polymer solution of OPT was investigated further using DSC analysis to understand the temperature-induced phenomena better. In addition, the binary polymer mixtures and single polymer solutions that contain each polymer component at the same concentration as found in OPT were also analyzed. The DSC thermograms are shown in Figure 4. As can be seen in Figure 4a, a broad endothermic peak was observed in the DSC thermogram of 20.000% (*w*/*w*) P407 (single P407). The broad endothermic signal corresponded to the micellization of P407 in water upon heating [10]. When the temperature is high enough, the PPO blocks of P407 become dehydrated and subsequently associate upon heating. The assembly of the dehydrated P407 blocks causes the micelle formation. Therefore, the DSC endotherm reflects the temperature and heat attributed to the micellization.

According to Figure 4b, there was no DSC peak observed for the other single polymer solutions, 0.595% (*w*/*w*) PCB (single PCB) and 7.349% (*w*/*w*) P188 (single P188). No thermal event found for the solution of P188 could be inferred that, in this condition, P188 concentration was below the critical micelle concentration (CMC) [39,40].

From Figure 4a, it can be seen that the mixture of 20.000% (*w*/*w*) P407 and 7.349% (*w*/*w*) P188 (binary P407/P188) exhibited only one endothermic peak which may correspond to the micellization of P407. The single endotherm observed in the present study is consistent with the one peak of micellization for the P407/P188 mixtures reported by Tipa et al. [40]. Only one peak of micellization observed for the binary P407/P188 mixtures seems to indicate that only P407 was able to form micelles because the concentration of P188 in the mixtures was lower than CMC or the micellization enthalpy of P188 at a certain concentration was too low to notice any peak of P188 micellization [40]. The shift of the endothermic peak to the lower temperature may be due to the change of the effective P407 concentration in the presence of another polymer [11]. The added P188 associates with water molecules and consumes water molecules for its hydration, reducing the accessible water molecules in the hydration layer around P407. The PPO blocks of P407 may be able to dehydrate and associate more easily upon heating, lowering the temperature required for micelle formation.

According to Figure 4, the binary mixture of P188 and PCB (binary P188/PCB) did not show any DSC peak (Figure 4b) while an overlapping double peak appeared in the DSC curve of binary P407/PCB mixture (binary P407/PCB in Figure 4a). The overlapping double peak was also observed in the DSC curve of OPT. The appearance of the overlapping endothermic peak could be attributed to two overlapping consecutive thermal events associated with the formation of two different micelle species [3]. Previous studies reported that the mixture of P407 and linear poly(acrylic acid) caused the formation of two different micelle species: pure 407 micelles and P407 micelles with attached polyacrylic acid [8,17]. The temperature-induced micellization of pure 407 micelles and P407 micelles with attached polyacrylic acid corresponds to the splitting of the endothermic process observed in the DSC curve. PCB is a lightly cross-linked polyacrylic acid polymer consisting of an acrylic acid backbone crosslinked with divinyl glycol. In this study, it is likely that the overlapping double peak could be caused by the temperature-induced micellization of pure micelles in co-existence with the micelles with attached PCB.

#### 3.2.2. Mucoadhesive Analysis

**Figure 5:**
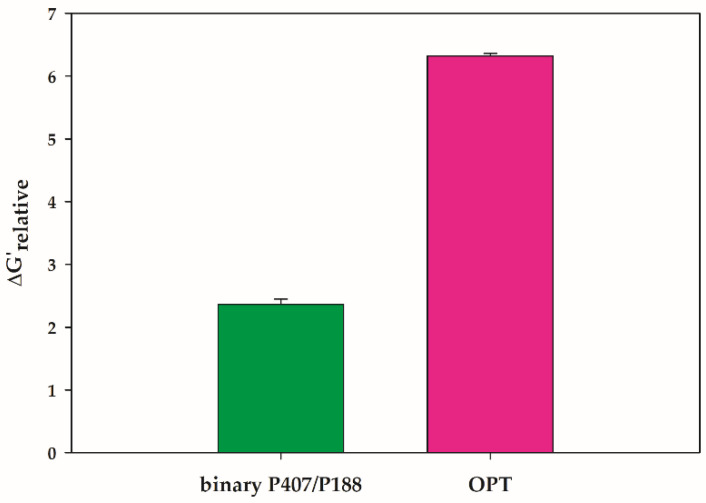
Relative rheological synergism (Δ*G*′*_relative_*) of OPT and the mixture of 20.000% P407 and 7.349% P188 (binary P407/P188).

The presence of mucoadhesive polymer in the composition may affect the mucoadhesive property of the composite hydrogels. The Δ*G*′*_relative_* value is indicative of the rheological synergism between the polymeric system and the mucin [27]. This rheological synergy can be utilized to predict the mucoadhesive characteristic of the material. There is no interaction between the polymer and the mucin if the Δ*G*′*_relative_* value equals one [41]. A higher Δ*G*′*_relative_* value reflects mucoadhesive associations between the polymeric gel and mucin [27,41]. The mucoadhesion of OPT was, therefore, assessed in terms of rheological synergy. Moreover, the mucoadhesive property of the binary polymer mixture composed of P407 and P108 at the same concentration as found in OPT was also analyzed. Figure 5 presents Δ*G*′*_relative_* for the binary mixture of 20.000% (*w*/*w*) P407 and 7.349% (*w*/*w*) P188 (binary P407/P188) and the OPT. Although positive values of Δ*G*′*_relative_* were observed for both samples, the OPT showed a considerably high value of Δ*G*′*_relative_* compared with that of the binary P407/P188. The presence of PCB in the OPT enhanced the rheological synergism of the polymeric system mixed with mucin. The strong rheological interaction between the polymeric blend and mucin due to the presence of PCB has also been reported for the mixture of P407 and PCB [26]. Mucoadhesion relies on the interpenetration and hydrogen bonding between polymers and mucus chains. Although the polymer chain of PCB is attached to the micelles, the polymeric system can interact with mucin due to the presence of certain carboxylic groups in the poly(acrylic acid) derivative [26]. As a result, polymeric hydrogels with available hydrogen-bonding groups are capable of forming strong bonds with mucin. Therefore, the OPT with good mucoadhesive characteristics, desirable complex viscosity, and optimal gelation temperature was considered for incorporating the model drug.

### 3.3. The Optimized Polymeric Blend with Incorporated Model Drug

MTZ is a hydrophilic compound that effectively inhibits anaerobic bacteria. MTZ has therapeutic potential for treating various local infections, such as periodontal disease and bacterial vaginitis [42,43]. Due to its hydrophilicity, MTZ is rapidly washed away by body fluid [42]. This limits drug exposure to the target site and makes MTZ less effective at treating infections. A potential strategy to overcome this limitation of MTZ is the use of a mucoadhesive in situ gel for local drug delivery to the infection cavity. Therefore, MTZ was selected as the model drug and incorporated into the OPT. The sample of OPT containing 0.75% (*w*/*w*) MTZ was referred to as OPT/MTZ.

#### 3.3.1. Rheological Behavior Comparison of OPT/MTZ and OPT

**Figure 6:**
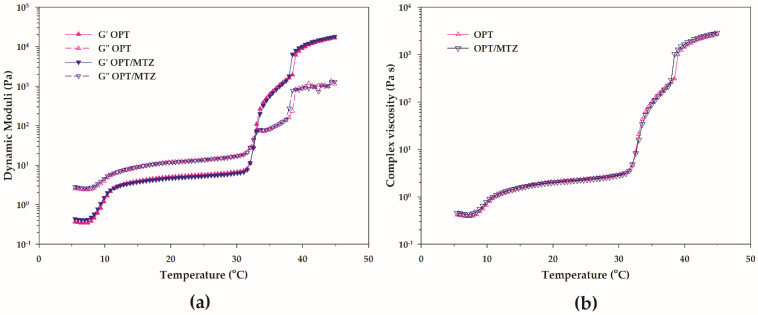
The temperature-dependent rheological characteristics of OPT and OPT/MTZ: (**a**) the plots of dynamic moduli as a function of temperature and (**b**) the plots of complex viscosity as a function of temperature.

The determination of temperature-dependent rheological behavior of hydrogels was necessary to investigate whether the presence of MTZ affects the heat-induced gelation of OPT. As can be seen in Figure 6, OPT/MTZ and OPT were thermosensitive gels. As can be seen in Figure 6a, OPT behaves like a solution (G″ > G′) at low temperature while transforming into a gel (G′ > G″) upon heating. The pure micelles in co-existence with the micelles with attached PCB formed upon heating as discussed in Section 3.2.1, and the existence of these micelle species may have contributed to the gradual increase in G′ and G″ following heating [3]. However, the sample retained its liquid-like behavior (G″ > G′) at low temperatures. Due to the ordered packing of micelles and intermicellar entanglements, both G′ and G″ grew significantly with further increasing temperature and then reached the crossover point (G′ = G″) reflecting the sol-gel transition. Complex viscosity values of OPT also ascended swiftly in the vicinity of the sol-gel transition point (Figure 6b). This trend was in general agreement with other studies, which reported that the phase transformation from a liquid to a semisolid gel led to an increase in the complex viscosity [4,44]. With the temperature increment, G′ became higher than G″ beyond the crossover point. Upon heating, the temperature-dependent viscoelasticity of OPT/MTZ followed the same pattern as OPT. In general, the crossover of G′ and G″ offered an estimate of the gelation temperature at which the system began to gel. The mean and standard deviation values of the gelation temperatures of OPT and OPT/MTZ were 33.45 ± 0.79 and 33.48 ± 1.65 °C, respectively. The mean and standard deviation values of the complex viscosity at 20 °C of OPT and OPT/MTZ were 1.98 ± 0.10 and 2.01 ± 0.03 Pa s, respectively. There was no statistical difference in the gelation temperatures (*p* = 0.196) and complex viscosity values (*p* = 0.125) of OPT and OPT/MTZ, which means that the addition of MTZ did not change the temperature-dependent characteristics of the polymer blend.

#### 3.3.2. In Vitro Release Study

**Figure 7:**
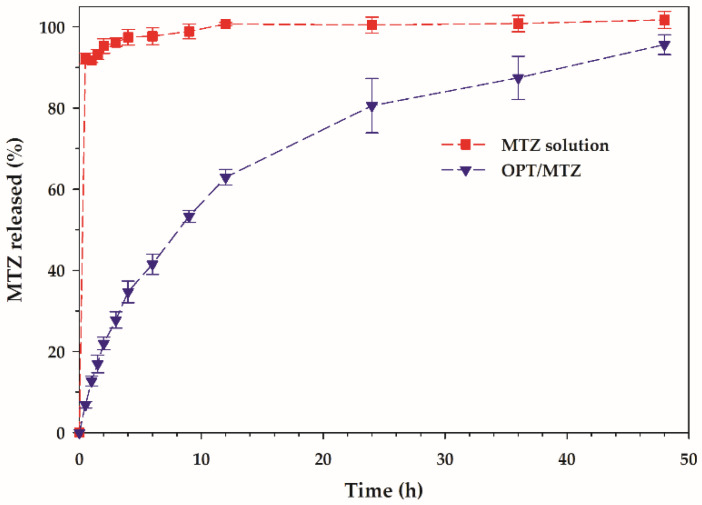
In vitro drug release profile of MTZ from MTZ solution and OPT/MTZ.

MTZ release profiles from OPT/MTZ and MTZ solution can be seen in Figure 7. It is observed that for the MTZ solution, almost all of the MTZ was released within 0.5 h. Therefore, the solubility and diffusion of MTZ was not the main determinant of release kinetics. The release of MTZ was sustained by the OPT composite hydrogel. The gel structure could act as a barrier, retarding the drug diffusion from the matrix to the outer medium. Not only the diffusion mechanism but also another mechanism could play a role in the drug release behavior. The MTZ release kinetic was then evaluated considering Higuchi, Hixson–Crowell and Korsmeyer–Peppas mathematical models.

For OPT/MTZ, the release parameters obtained from the fitting of the experimental data are given in Table 3. The coefficient of determination (R^2^) is useful for determining the most suitable model [30]. The best fit was found to be the Korsmeyer–Peppas mathematical model with *n* value = 0.63 (R^2^ = 0.9929), indicating that the drug release was modulated by an anomalous transport mechanism. The anomalous transport involves a coupling of Fickian diffusion and polymer relaxation. During the test in this condition, only one side of the gel was exposed to the dissolution medium, so the geometry of the matrix was thought to be a slab [30]. The MTZ release data was then fitted to the Peppas–Sahlin model to assess the contribution of Fickian diffusion and relaxation processes [45].

(9) $$Q_{t}=k_{1}t^{0.5}+k_{2}t$$

The Peppas–Sahlin model describes the drug release in terms of diffusion-controlled (*k*_1_*t*^0.5^) and relaxation-controlled transport (*k*_2_*t*) processes. The *k*_1_ and *k*_2_ values obtained from the curve fitting (R^2^ = 0.9890) were 11.992 and 2.043, respectively. The *k*_1_/*k*_2_ ratio was 5.870, indicating that the diffusion mechanism was predominantly responsible for MTZ release from the OPT composite matrix.

## 4. Conclusions

A Box–Behnken design was used to investigate the effects of P407, P188 and PCB on the temperature-dependent rheological properties, including complex viscosity in the liquid stage and gelation temperature. The optimization using the Box–Behnken design was successfully applied to maximize PCB content in the blend while achieving the preferable temperature-dependent characteristics. The optimized P407/P188/PCB blend was the mixture of 20.000% (*w*/*w*) P407, 7.349% (*w*/*w*) P188, and 0.595% (*w*/*w*) PCB. Pure micelles coexisting with micelles with attached PCB arose during the heating process. In addition, the blend achieved a higher degree of mucoadhesion by incorporating PCB into the P407/P188 matrix. A model hydrophilic drug, MTZ, was introduced into the optimal hydrogel. The in vitro drug release study demonstrated that the gel matrix sustained MTZ release, which was regulated by an anomalous mechanism. The optimal ternary P407/P188/PCB hydrogel could be used as a potential platform for mucoadhesive in situ gelling drug delivery.

## Figures and Tables

**Table 1:** Variables and Box–Behnken design.

**Independent Variables**		**Levels**	
	**Low (−1)**	**Medium (0)**	**High (+1)**
X_1_: P407 concentration (%*w*/*w*)	20	21.5	23
X_2_: P188 concentration (%*w*/*w*)	3	9	15
X_3_: PCB concentration (%*w*/*w*)	0	0.3	0.6
	**Desirability Constraints**		
Gelation temperature (°C)	33–36		
Complex viscosity (Pa s)	<2		
PCB concentration (%*w*/*w*)	Maximum		
**Run Number**	**Levels of Independent** **Variables**		
	X_1_	X_2_	X_3_
1	0	0	0
2	−1	0	−1
3	0	0	0
4	0	+1	+1
5	0	0	0
6	0	−1	+1
7	−1	0	+1
8	−1	−1	0
9	−1	+1	0
10	0	−1	−1
11	+1	0	−1
12	0	0	0
13	0	0	0
14	0	+1	−1
15	+1	−1	0
16	+1	0	+1
17	+1	+1	0

**Table 2:** The predicted and observed responses of the optimized thermosensitive hydrogel.

Responses	Predicted Value	Experimental Value	95% Confidence Interval (Lower-Upper)
Gelation temperature (°C)	33.00	33.45 ± 0.79	31.62–34.38
Complex viscosity at 20 °C (Pa s)	1.990	1.977 ± 0.10	1.861–2.119

**Table 3:** Release parameters for hydrogel according to different mathematic models.

	Higuchi		Hixson–Crowell		Korsmeyer–Peppas		
	*K_H_*	R^2^	*K_HC_*	R^2^	*n*	*K_KP_*	R^2^
OPT/MTZ	16.601	0.9593	0.029	0.9122	0.63	13.604	0.9929

## Data Availability

Not applicable.

## Supplementary Materials

The following supporting information can be downloaded at: https://www.mdpi.com/article/10.3390/polym14091836/s1, Figure S1: Calibration curve of metronidazole.
