# Supplementary material for: Thermosensitive Polymer Blend Composed of Poloxamer 407, Poloxamer 188 and Polycarbophil for the Use as Mucoadhesive In Situ Gel
Source: Polymers (Basel). 2022 Apr 29;14(9):1836. doi: 10.3390/polym14091836 (PMC9102451; doi:10.3390/polym14091836)
Supplement: Supplementary file 1 [file polymers-14-01836-s001.zip › polymers-1688663-supplementary.pdf]

polymers

Supplementary Material: Thermosensitive Polymer Blend Composed of Poloxamer 407, Poloxamer 188 and Polycarbophil for the Use as Mucoadhesive In Situ Gel

Namon Hirun, Pakorn Kraisit and Vimon Tantishaiyakul

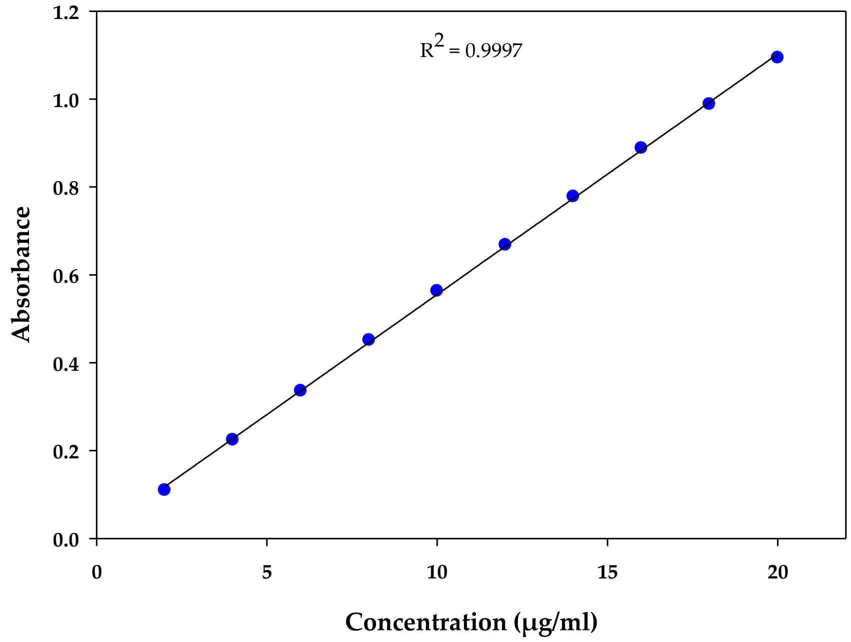

Figure S1. Calibration curve of metronidazole.
